# Functional performance of school children diagnosed with developmental delay up to two years of age

**DOI:** 10.1016/j.rppede.2015.10.001

**Published:** 2016

**Authors:** Lílian de Fátima Dornelas, Lívia de Castro Magalhães

**Affiliations:** a Prefeitura Municipal de Uberlândia, Uberlândia, MG, Brazil; b Departamento de Terapia Ocupacional, Escola de Educação Física, Fisioterapia, Educação Física (EFFTO), Universidade Federal de Minas Gerais (UFMG), Belo Horizonte, MG, Brazil

**Keywords:** Evaluation, Child development, Students

## Abstract

**Objective:**

To compare the functional performance of students diagnosed with developmental delay (DD) up to two years of age with peers exhibiting typical development.

**Methods:**

Cross-sectional study with functional performance assessment of children diagnosed with DD up to two years of age compared to those with typical development at seven to eight years of age. Each group consisted of 45 children, selected by non-random sampling, evaluated for motor skills, quality of home environment, school participation and performance. ANOVA and the Binomial test for two proportions were used to assess differences between groups.

**Results:**

The group with DD had lower motor skills when compared to the typical group. While 66.7% of children in the typical group showed adequate school participation, receiving aid in cognitive and behavioral tasks similar to that offered to other children at the same level, only 22.2% of children with DD showed the same performance. Although 53.3% of the children with DD achieved an academic performance expected for the school level, there were limitations in some activities. Only two indicators of family environment, diversity and activities with parents at home, showed statistically significant difference between the groups, with advantage being shown for the typical group.

**Conclusions:**

Children with DD have persistent difficulties at school age, with motor deficit, restrictions in school activity performance and low participation in the school context, as well as significantly lower functional performance when compared to children without DD. A systematic monitoring of this population is recommended to identify needs and minimize future problems.

## Introduction

Developmental delay (DD) is a condition in which the child is not developing and/or does not achieve skills consistent with what is expected for their age.^1^ Although the term “delay” gives the impression of a relatively benign condition that improves with age, many of those children do not receive follow-up with systematic assessments and have problems at school age and adult life.^2^ In fact, it is estimated that 60–70% of children born with risk conditions will require support from special education services in elementary and high school, and there is evidence that gaps in the development of children about to enter school may impair their school performance and future opportunities.^3^

Studies^4^^,^ 5 on the development outcome at school age indicate that DD has an effect on a complex range of symptoms, without a defined disease profile, and thus it is important to obtain information about what the child is capable of doing in a daily context, to better understand its consequences. Although it is recommended that the use of the term “DD” be restricted to the first five years of life,^6^ in Brazil its use is common throughout childhood and adolescence, without a better understanding of the development outcome of these children, especially regarding functional performance in the school context. We should therefore investigate the outcome of these children in terms of final diagnosis, as well as the impact of the delay on the functional and academic performance.

As explained by the International Classification of Functioning, Disability and Health–ICF–WHO (World Health Organization),^7^ in order to understand the impact of a health condition such as DD on the child's life, it is important to perform an extensive assessment to obtain information not only about basic body functions, but also on the activity and participation in different contexts. In this study, the ICF–WHO model was used to guide the process of assessing children with a history of DD and describe the child's performance in the school context. The aim of the study was to compare the functional performance of students who were diagnosed with DD up to two years of age, with that of peers with typical development.

## Method

This was a cross-sectional study to evaluate the functional performance of students who were diagnosed with DD up to two years of age and those with typical development at seven to eight years of age, selected by non-random sampling and matched for age, gender and family income. Each group consisted of 45 children and the subjects with DD were recruited from Associação de Assistência à Criança Deficiente de Minas Gerais (AACD/MG); their peers were selected from the same schools where the children from the first group studied.

AACD/MG is specialized in treating individuals with physical disabilities. Babies who have pre-, peri-, and postnatal complications and/or developmental problems are referred by physicians from basic health units or hospitals and by the parents, being evaluated by the AACD/MG team, who verify the need for intervention. The diagnosis of DD is based on a clinical assessment carried out by the physician of the institution, through neurological assessment. As the term “DD” is not found in the ICD-10, in order to be treated at the institution, these children are classified according to the categories of Chapter VI (Nervous System Diseases) – code: G00-G99, specifically in the subcategory closer to the term, Unspecified Cerebral Palsy – code: G80.9. After medical assessment, children are referred to overall assessment, in which the multidisciplinary team, consisting of a physiotherapist, speech therapist, psychologist and occupational therapist, makes the direct clinical observation, describes the child's development, discusses the case with the physician and defines whether the child will benefit from intervention and what therapies are necessary. All children admitted to the AACD undergo an initial medical evaluation, as well as a final evaluation, when they meet the criteria for discharge. Assessments are predominantly clinical, without the use of standardized tests. Children with DD admitted at AACD usually exhibit motor delay and have weekly consultations with physical therapy, hydrotherapy and occupational therapy professionals to acquire typical gait, when they are discharged from care. Even though the AACD/MG does not provide a longitudinal follow-up program for these children, as they have no physical disability, they may return to the institution to receive recommendations or to undergo therapies with specific goals, albeit in the short term. There is no specific follow-up program for this population, and the return visits depend on the parents’ decision.

**Figure 1:**
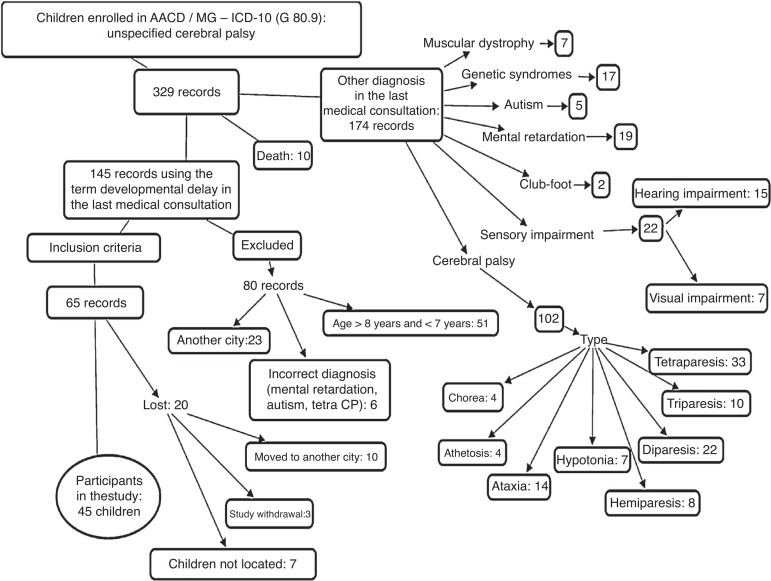
Screening of patients’ records to select the participants in the DD group.

In this context, eligible children for the study were identified through the review of medical files with the G80.9 code from ICD-10, from August 2001 to August 2009, in the AACD/MG filing sector. The initial list contained 329 records that were screened, according to the inclusion and exclusion criteria, until 45 study participants were obtained, as shown in Fig. 1. The inclusion criteria were children of both genders, born between January 2003 and April 2006, living in the city of Uberlândia (MG), diagnosed with DD, which had pre-, peri-, and postnatal complications, with no evident neurological and/or orthopedic disorders, malformations, as well as visual or hearing impairments. The study only included children who had, in the last medical evaluation, typical gait, the ones who were discharged from therapeutic care, attended school regularly and whose parents or guardians signed the informed consent form (ICF), authorizing their participation in the study. Children were excluded if their diagnosis changed to cerebral palsy, muscular dystrophy, autism, mental retardation or syndromes, as well as those who remained with a diagnosis of DD, but had evident neurological and/or orthopedic disorders, malformations, and visual or hearing impairment.

The children with typical development were recruited from the same schools the cases from AACD/MG attended. The recruitment of each child from the typical group was carried out in the same classroom of each child diagnosed with DD. The authors, after receiving parental consent of the child with DD, went to the school and made contact with the teacher. Each teacher was asked to identify, in the roll call, students of the same gender and age as those diagnosed with DD. After the children were identified, they were chosen by drawing lots, and an invitation letter (ICF) was sent to the parent/guardian. Along with the ICF, the parent/guardian answered a brief questionnaire about the development history in order to ensure that the child fit the group profile. Only children whose ICFs were returned with the parents’/guardian's signature and with the questionnaire answered by parents/guardians were evaluated; otherwise, the teacher chose another student by drawing lots. Children were excluded if they had, according to the questionnaire, a diagnosis of specific neurological or genetic disorders and risk factors such as prematurity and/or low birth weight, hearing and visual impairments, as well as orthopedic problems (fracture of the lower limbs and others), continuous use of anticonvulsants, prolonged illness in the three months prior to the test, history of grade repetition and difficulties that required pedagogical support or some kind of specialized therapy (physical therapy, speech therapy, psychological support, occupational therapy).

The following tools were used in the research:

- **Semi-structured system** for data extraction from records of DD children from the AACD/MG. Information was obtained about the child's history (gestational age, birth weight and neonatal conditions), medical diagnosis on entering the institution and the one registered in the last consultation in the unit, parents/guardian information (maternal and paternal schooling, family income), school data (name, address, telephone, teacher and grade), as well as rehabilitation aspects (child's initial and final evaluations made by the rehabilitation team and the therapies performed during the intervention period). As the initial and final evaluations of each child is made descriptively, the information was categorized into three components: (a) motor, for the evaluation of the physical therapy sector; (B) activity, for the occupational therapy sector, and (c) participation, for the psychology sector assessment; the information of each component was coded to indicate whether the child's developmental level was delayed, suspected or appropriate. Data collection from medical records was performed by two researchers, who had been previously trained with 20 records, obtaining a good Kappa agreement index (0.63–0.79).
- **Academic performance rating:** as there is no grading system in the assessed age range, a classification was created based on the information obtained from the teacher on the grading that the child received regarding the skills worked throughout the school year. According to the information obtained from the schools, the children are assessed throughout the year by the teacher, who assigns grades to the acquisition of skills and identifies the level the children are at in reading and writing skills (alphabetical, syllabic-alphabetic, syllabic and pre-syllabic). This information was combined and categorized as follows: (a) Level 1 – consolidated acquisition of the expected skills for the school year – the child showed alphabetic level and/or grade A, (b) Level 2 – developing acquisition of the expected skills for the school year – syllabic-alphabetic or syllabic level and/or grade B, and (c) Level 3 – no mastery of the skills expected for the school year – presented pre-syllabic level and/or grading.
- **Movement Assessment Battery for Children – MABC-2** 8: It is a standardized test to identify motor coordination disorders in children aged 4–16 years old, divided into three areas: manual dexterity, hand grasping, throwing and balance. The sum of scores for each category provides a standardized score, and the sum of the three categories provides the total score, which is converted to percentile. A cut-off ≤15% indicates possible motor impairment, and a score ≤5% indicates definitive motor deficits. Children with score ≤5% were considered as having motor coordination problems or signs of Developmental Coordination Disorder (DCD); a score of 6–15%, suspected cases, and children with a score >15% were considered as having normal motor performance.
- **School Function Assessment – SFA** 9: This is a questionnaire to assess functional performance and the participation of children aged 5–12 years in the school environment, consisting of three parts: participation in different school environments, assistance with tasks and task performance. The raw SFA scores are converted into a scale of 0–100, with the latter being the highest point or a fully operational degree in the assessed area. SFA results can be interpreted in two ways, at the basic and advanced levels. For this study, we used the basic level, which shows if the child's function in the school environment is as expected for children of the same age and at the same school year.
- **Family Environment Resource Inventory – FERI** 10: It is a questionnaire used to assess resources of the family environment, divided into three areas: material resources, activities that signal family life stability and parenting practices. To obtain the relative score in 10 points, the following formula was used: raw score/topic maximum score ×10, in which the raw score is the number of checked items and the maximum score is the total number of items, except in topics 8–10, which have specific scores. The relative score is useful to compare the scores between inventory items.

Data were collected from January 2010 to January 2012. All children who participated in this study were evaluated by MABC-2 test, the SFA and FERI questionnaires, and a classification of school performance was made based on the teachers’ reports. The MABC-2 is one of the most widely used tests in research for the diagnosis of DCD and was used in this study to evaluate the children's motor development. The MABC-2 has good levels of test-retest (0.75) and inter-rater reliability (0.70)^11^^,^ 12; it has been used in different countries and there is evidence of score validity for Brazilian children.^13^ As MABC-2 is a performance test, the inter-rater reliability was verified before data collection, yielding an index of 0.80 (Intraclass Correlation).

SFA has been used in Brazil and it is a questionnaire that is easy to apply, of which content is considered adequate to document the functional profile of school-aged children. Although it has not been validated and standardized for Brazilian children, North-American studies support the validity and reliability of this tool.^14^ The FERI has shown to be useful to differentiate family environment characteristics of children with different levels of school performance and behavior problems and has appropriate parameters of test-retest reliability (0.92–1.00), as well as good internal consistency (0.84).^15^ Although this inventory does not have a cut-off, it has been used in Brazil to compare groups of children.

All children lived in the city of Uberlândia (MG), and all were evaluated by the first author, previously trained to apply the tests and questionnaires. The SFA questionnaire and information about school performance (grading) were applied together with the child's teacher in the child's school. On that visit, the first author explained to the teacher about the drawing of one child's name in the same classroom the child with DD attended. These children were subsequently evaluated after they brought the signed ICF.

The first author visited 35 schools (20 municipal schools, eight state schools, six private and one federal school) as, of the 45 children from the DD group, only ten students attended the same school. To enter the municipal schools, the researcher had to obtain the consent from the Education Secretariat of the Uberlândia Municipal Government. In the other schools (state, federal and private), individual contact was made, and all schools received the documentation demonstrating that the children with DD had their parents’ permission (ICF) and the study had the approval from the Institutional Review Board of AACD (No. 09/2010) and the Institutional Review Board of Universidade Federal de Minas Gerais (COEP/UFMG No. ETIC 0482.0.203.000-10).

The Statistical Package for Social Sciences (SPSS) for Windows, version 17.0, was used for data analysis. The description of the groups was made by measures of central tendency (mean and standard deviation) or frequency. The Shapiro-Wilk's normality test was applied, which found that most of the variables had a normal distribution and thus, parametric tests were chosen. Analysis of Variance (ANOVA) was used for inferential statistics, aiming to identify possible differences between the groups DD and typical development regarding the quantitative variables of motor performance, environmental resource and participation at school. The Binomial test for two proportions was used for the categorical variable performance in the academic content. A significance level ≤0.05 was considered for all analyses.

## Results

Of the 65 children from AACD/MG diagnosed with DD, 45 (69.3%) participated in the study, whereas the others (20; 30.7%) were lost due to study withdrawal, moving to another city and children not being found. Therefore, the DD group consisted of 45 children diagnosed with DD, and the typical group consisted of 45 children with typical development, selected by non-random sampling, matched by gender, age and household income. Table 1 shows the descriptive information of the groups.

**Table 1:** Characteristics of children with delayed neuropsychomotor development (DD) and typical development at school age.

Characteristics of participants		Groups	
		With DD	Typical development
*Gender* *			
	Male	26 (57.8)	26 (57.8)
	Age (months) **	95.8±7.7 a	95.4±7.6 a

*Family income* *			
	<3 MW	27 (60%) b	26 (57.7%) b
	3–5 MW	7 (15.6%) c	12 (26.7%) c
	>5 MW	11 (24.4%) d	7 (15.6%) d
	Gestational age	35.49 (4.88) **	>38 weeks
	Mean birth weight **	2.289±1116.17	3.086±500.37

*Maternal schooling* *			
	College/University	11 (24.4)	13 (28.9)
	High School	20 (44.4)	25 (55.6)
	Elementary School	13 (29)	6 (13.3)
	Illiterate	1 (2.2)	1 (2.2)

a *p* =0.794.

b *p* =0.830.

c *p* =0.197.

d *p* =0.292.

* Frequency (percentage) of children in each category.

** Mean ± standard deviation.

MW, minimum wages.n=45 in each group.

In the DD group, 23 (51.1%) children were born prematurely, with gestational age ranging from 24 to 36 weeks; in the neonatal period, 17 (37.8%) had jaundice, 13 (28.9%) had seizures, 14 (31.1%) reported the need for oxygen supplementation, and 12 (26.7%) had signs of perinatal hypoxia. The typical development group consisted of children born at term, with no record of relevant neonatal complications.

The children from the DD group commonly had, as initial development characteristics, motor delay (24; 55.6%) and suspected level of development in the areas of activity (27; 60%) and participation (27; 60%). The end of the multidisciplinary intervention, which lasted on average 2.61±1.96 years, was mainly due to the fact that motor development had been considered appropriate for age (41; 91.1%).

There was a mean difference between the groups with statistical significance in all MABC-2 areas (Table 2). The DD group had lower performance in all test domains, with most children from this group (28; 62.2%) showing motor difficulty, while four (8.9%) were at risk for motor difficulty. In the typical group, five (11.1%) children had motor difficulty and six (13.3%) were at risk for motor difficulty.

**Table 2:** Comparisons between percentiles of motor performance for the groups with developmental delay (DD) and typical development.

MABC-2	Mean ± SD			Minimum–maximum		*p* -value a
	With DD	Typical		With DD	Typical	
Manual dexterity	20.1±26.2	48.7±31.2		0.5–98	2–99.9	<0.001
Throwing and grasping	19.8±21.2	30±23.5		0.5–91	1–91	0.034
Balance	14±21.8	33.2±26		0.1–95	2–99	<0.001
Total motor	13.2±21.6	34.3±27		0.1–91	2–98	<0.001

a ANOVA.

MABC-2, movement assessment battery for children; SD, standard deviation; n, 45 in each group.

In the SFA, children from the DD group had lower mean scores in all assessed questionnaire items when compared to the typical group, and only the self-care category (*p*=0.183) showed no difference between groups. While 30 children from the typical group (66.7%) showed effective participation in the school environment, with help in cognitive and behavioral tasks similar to that offered to classmates of the same year, only 10 (22.2%) children with DD showed the same performance. Regarding academic activities, the typical development group showed a consistent, superior performance in all tasks when compared with the DD group. Children from the DD group had limited performance, especially in activities that required positive interaction (26; 57.8%), behavioral control (26; 57.8%) and completing tasks (28; 62.2%) (Table 3).

**Table 3:** Comparative data in the school participation questionnaire score for groups with developmental delay (DD) and typical development.

SFA		Mean ± SD			Minimum–Maximum		*p* -value b
		With DD a	Typical a		With DD	Typical	
*Part I: participation*							
	Participation in school environment	80±15.8	94.7±0.6		54–100	70–100	<0.001

*Part II: help with tasks*							
	Cognitive and behavioral	75.5±23.3	90.5±12.8		0–100	53–100	<0.001

*Part III: activity performance*							
	Use of materials	84.7±17.4	95.3±8.9		54–100	68–100	<0.001
	Written work	77.5±23.5	91.8±11.2		15–100	64–100	<0.001
	Functional communication	82.0±20.3	93.2±12.7		30–100	49–100	0.002
	Memory and comprehension	80.7±19.7	93.3±15.0		39–100	27–100	0.001
	Safety	87.0±20.4	98.2±7.6		40–100	53–100	0.001
	Self-care	92.9±16.9	96.9±10.1		28–100	55–100	0.183
	Positive interaction	73.3±22.0	90.7±11.7		15–100	54–100	<0.001
	Behavioral control	65.8±29	85.9±15.9		0–100	48–100	<0.001
	Following rules	76.4±24.0	91.8±12.4		0–100	55–100	<0.001
	Obeying adults’ orders	78.0±22.0	87.7±17.8		29–100	10–100	0.024
	Behavior and task completion	71.4±21.9	89.7±11.8		28–100	59–100	<0.001

a Mean raw data transformed to a scale of 0–100.

b ANOVA.

SFA, school function assessment; SD, standard deviation; n, 45 in each group.

Regarding academic performance, there were differences between the groups, with statistical significance at levels I (*p*=0.001) and II (*p*=0.008). Most (38; 84.5%) of the children from the typical development group showed mastery of the academic content (level I – alphabetic and/or excellent grading), and the rest (6, 13.3% – level II: syllabic-alphabetic or syllabic, good grading; 1, 2.2% – level III: pre-syllabic and/or fair grading) were in the development phase. In the DD group, most (24; 53.3%) achieved level I and level II (17; 37.8%), with only four (8.9%) children still receiving fair grading.

As indicated in Table 4, although there is a difference in the means between groups regarding the FERI items, only two indicators reached statistical significance (*p*≤0.05). In the DD group, children were less active in their free time and shared fewer activities with their parents at home.

**Table 4:** Comparative data of the Family Environment Resource Inventory (FERI) for groups with developmental delay (DD) and typical development.

FERI	Raw score Mean ± SD			Relative score a Mean ± SD		*p* -value b
	With DD	Typical		With DD	Typical	
Diversity of activities in free time	3.8±1.2	4.4±0.9		6.2±1.9	7.4±1.6	0.003
Trips made in the last 12 months	7.4±2.3	8.2±2.9		4.1±1.3	4.9±1.6	0.121
Regular scheduled activities	1.3±1.4	1.8±1.9		1.6±1.7	2.2±2.4	0.186
Activities with parents at home	7.4±2.2	8.8±0.6		7.4±2.2	8.8±1.6	0.001
Toys and other materials	13.2±3.2	13.4±3.4		7.3±1.8	7.4±1.9	0.775
Diversity of magazines and newspapers	4.9±2.8	5.3±2.3		5.4±3.1	5.9±2.5	0.430
Diversity of books	5.5±1.6	5.4±1.6		6.8±2.0	6.7±2.0	0.809
School work supervision	11.6±2.4	12.6±2.2		6.4±1.3	7.0±1.2	0.058
Daily routine with a well-defined schedule	10.9±3.9	10.6±3.5		6.8±2.5	6.6±2.2	0.720
Moments when the family gets together	8.3±2.4	8.7±2.2		6.9±2.0	7.3±1.8	0.368
Total FERI	74.6±10.6	79.2±13.6		6.0±0.9	6.4±1.1	0.081

a Relative score: mean raw data transformed to a scale from 0 to 10.

b ANOVA, relative score.

SD, standard deviation; n, 45 in each group.

## Discussion

Although the term DD is widely used in Brazilian literature, little is known about the outcome of these children. This study demonstrates that children with a diagnosis of DD attained up to two years of age show, at school age, motor limitations, restrictions in school activity performance, low participation in the school context and significantly lower functional performance when compared to the children without a history of delay. Although children persist with the delay, the functional outcome was better than the motor one, suggesting the possibility of adaptation, which is consistent with the WHO–ICF perspective^7^ that the association between motor function and participation is not a linear one. Environmental stimuli, which can act as a protective factor, do not seem to influence these results.

It is known that the risk factors for delay are multiple and the accretion of conditions can determine a higher impact on child development.^16^^,^ 17 Children from the DD group included in this study came from low-income families, with most families receiving less than three minimum wages, and the majority of the mothers had only high-school level education. Additionally, 51.1% of children in the DD group had a history of prematurity, low birth weight and neonatal neurological complications. Although one cannot exclude the effect of other factors not assessed on the outcome of the DD group, biological risk, represented especially by prematurity, was decisive on the other factors. As discussed by some authors,^18^^,^ 19 in spite of the low investment in determining the etiology, many studies indicate biological factors as determinants of most DD cases. In the study of Srour et al.,^20^ for instance, which investigated the cause of the delay through clinical and laboratory tests, the etiology of 77% of the cases was identified, and brain malformation, hypoxic-ischemic encephalopathy and chromosomal abnormalities were the most common causes.

The high frequency (62.2%) of motor disorders at school age found in the DD group corroborates the literature. A meta-analysis by Williams et al.,^21^ including studies on school children born prematurely, indicated a prevalence of up to 40.5% of motor alterations versus 6% in the general population. Although the typical group also includes children with motor difficulties (11.1%), the frequency was close to that expected for the general population, as observed in the study by Goyen and Lui^22^ who, when assessing preterm and full-term infants with the MABC-2 test, found a prevalence of motor deficit of 42% in the preterm and 8% in the full-term infants.

As for school performance, measured by the academic content domain according to the teacher's assessment, it was observed that just a little over half (53.3%) of the children from the DD group had excellent grading, indicating advancement in the literacy process. It is worthy noting that, even in the presence of motor alterations, as mentioned before, these children achieved mastery of the academic content. Even though the children persisted with the delay, the functional outcome was better than the motor one, suggesting that children are able to adapt or that it is possible to modify the environment to facilitate participation and learning.^5^

Children from the DD group, however, had worse scores in all areas of school participation in the SFA and only 22.2% of them, versus 66.7% in the typical development group, effectively participated in the school environment, without the need for extra help in cognitive and behavioral tasks. Riou et al.^5^ also found that only 17% of children with DD participated in the classroom without help. Possibly, the motor delay, as recorded in this study, had more impact on the performance of the necessary activities to participate in class (e.g. handling materials, written work) than on academic performance measured by literacy, which does not necessarily require the motor component.

The study's limitations include the use of imported tests, without fully validated cut-offs for the Brazilian population, using the teachers’ reports to classify the academic performance, and the fact that it is a convenience sample. Foreign tests have been routinely used in Brazilian studies, and the MBC-2 was recently validated.^13^ Additionally, care was taken to collect comparative data. As many children in the DD group had literacy problems, it would be important to perform a language assessment, which should be included in future studies. Teachers’ reports were used in other studies, such as the one by Pritchard et al.,^23^ which, when comparing the qualitative teacher's evaluation with standardized measurements, found that the report detected two to three times more children likely to have learning difficulties. It is recommended that future studies leave the rehabilitation center context to include a broader population from basic health units, in order to investigate the outcome of milder and more varied cases of DD, as it can provide necessary statistical power to detect differences in environmental factors.

This study, performed in a clinical setting, emphasizes the importance of recognizing the need for a longitudinal follow-up on the development of children who receive an early diagnosis of DD as, for many of them, the delay is a sign of future conditions that should be better monitored and diagnosed as early as possible. The monitoring should be performed at least until school age, in order to identify the needs as they arise, minimizing problems at school age and adulthood. Sporadic evaluations can help professionals and parents understand what is happening, so adequate support can be provided to the child up to the “final diagnosis” definition, as the “DD” term should be used only as a temporary diagnosis.
