# Supplementary material for: Functional performance of school children diagnosed with developmental delay up to two years of age
Source: Rev Paul Pediatr. 2016 Jan-Mar;34(1):78–85. doi: 10.1016/j.rppede.2015.10.001 (PMC4795725; doi:10.1016/j.rppede.2015.10.001)
Supplement: Translation

# Translation: Desempenho funcional de escolares que receberam diagnóstico de atraso do desenvolvimento neuropsicomotor até os dois anos

**Authors:** Lílian de Fátima Dornelas, Lívia de Castro Magalhães

**Affiliations:** a Prefeitura Municipal de Uberlândia, Uberlândia, MG, Brasil; b Departamento de Terapia Ocupacional, Escola de Educação Física, Fisioterapia, Educação Física (EFFTO), Universidade Federal de Minas Gerais (UFMG), Belo Horizonte, MG, Brasil

**Keywords:** Avaliação, Desenvolvimento infantil, Escolares

## Abstract

**Objetivo:**

Comparar o desempenho funcional de escolares que receberam diagnóstico de atraso do desenvolvimento neuropsicomotor (ADNPM) até dois anos com pares com desenvolvimento típico.

**Métodos:**

Estudo transversal com avaliação do desempenho funcional em crianças que receberam diagnóstico de ADNPM até os dois anos e em crianças com desenvolvimento típico nas idades de sete e oito anos. Cada grupo foi constituído por 45 crianças, selecionadas por amostragem não aleatória, avaliadas quanto à coordenação motora, qualidade do ambiente familiar, participação e desempenho na escola. Os testes Anova e binomial para duas proporções foram usados para verificar diferença entre os grupos.

**Resultados:**

O grupo com ADNPM obteve desempenho motor inferior quando comparado com o grupo típico. Enquanto 66,7% das crianças do grupo típico tiveram participação adequada na escola, receberam auxílio nas tarefas cognitivas e comportamentais similar ao oferecido às demais crianças do mesmo nível, apenas 22,2% crianças com atraso apresentaram o mesmo desempenho. Embora 53,3% das crianças com atraso tenham atingido desempenho acadêmico esperado para o nível escolar, houve limitações em algumas atividades. Apenas dois indicadores do ambiente familiar, diversidade e atividade com os pais em casa mostraram diferença estatisticamente significativa entre os grupos, com vantagem para o grupo típico.

**Conclusões:**

Crianças com ADNPM apresentam dificuldades persistentes na idade escolar, com déficit motor, restrições no desempenho de atividades escolares e baixa participação no contexto escolar, além de desempenho funcional significativamente inferior ao de crianças sem história de atraso. Recomenda-se o acompanhamento sistemático dessa população para identificar necessidades e minimizar problemas futuros.

## Introdução

O atraso do desenvolvimento neuropsicomotor (ADNPM) é uma condição na qual a criança não se desenvolve e/ou não alcança habilidades de acordo com o que é esperado para sua idade.^1^ Apesar de o termo “atraso” passar a impressão de ser uma condição relativamente benigna, que melhora com a idade, muitas dessas crianças não recebem acompanhamento com avaliações sistemáticas e vêm a apresentar problemas na idade escolar e vida adulta.^2^ De fato, estima-se que 60-70% das crianças que nascem em condições de risco exigirão apoio dos serviços de educação especial no ensino fundamental e médio, há evidências de que lacunas no desenvolvimento de crianças que estão prestes a entrar na escola podem comprometer o desempenho escolar e as oportunidades no futuro.^3^

Estudos^4^^,^ 5 sobre o desfecho do desenvolvimento na idade escolar apontam que o ADNPM repercute em uma gama complexa de sintomas, sem perfil nosológico definido, é importante obter mais informações sobre o que a criança é capaz de fazer no contexto cotidiano, para entender melhor suas consequências. Embora seja recomendado que o uso do termo ADNPM restrinja-se aos primeiros cinco anos de vida,^6^ no Brasil é comum o seu uso ao longo da infância e adolescência, sem se conhecer melhor o desfecho do desenvolvimento dessas crianças, especialmente no que concerne ao desempenho funcional no contexto escolar. Deve-se, portanto, investigar o desfecho dessas crianças, tanto em termos do diagnóstico final como do impacto do atraso no desempenho funcional e acadêmico.

Conforme explicitado pela Classificação Internacional de Funcionalidade, Incapacidade e Saúde (CIF), da Organização Mundial de Saúde,^7^ para entender o impacto de uma condição de saúde como o ADNPM na vida da criança é importante fazer avaliação ampla, para obter informações não só sobre funções básicas do corpo, mas também sobre a atividade e participação nos diferentes contextos. No presente estudo, o modelo da CIF foi usado para guiar o processo de avaliação de crianças com história de ADNPM e descrever o desempenho da criança no contexto escolar. O objetivo do estudo foi comparar o desempenho funcional de escolares que receberam diagnóstico de ADNPM até os dois anos com pares com desenvolvimento típico.

## Método

Estudo transversal com avaliação do desempenho funcional de escolares que receberam diagnóstico de ADNPM até os dois anos e com desenvolvimento típico, nas idades de sete e oito anos, selecionadas por amostragem não aleatória e pareados quanto à idade, ao sexo e à renda familiar. Cada grupo foi constituído por 45 crianças. Os portadores de ADNPM foram recrutados na Associação de Assistência à Criança Deficiente de Minas Gerais (AACD/MG) e os seus pares foram selecionados nas mesmas escolas em que estudavam as crianças do primeiro grupo.

A AACD/MG é especializada no atendimento a pessoas com deficiência física. Bebês que apresentam intercorrências pré/peri/pós-natal e/ou problemas no desenvolvimento são encaminhados pelos médicos dos postos de saúde ou dos hospitais e pelos próprios pais e avaliados pela equipe da AACD/MG, que verifica se há recomendação para intervenção. O diagnóstico de ADNPM é baseado na avaliação clínica feita pelo médico da Instituição, por meio da semiologia neurológica. Como esse termo não consta da CID-10, para que essas crianças possam ser atendidas na Instituição elas são enquadradas nas categorias do Capítulo VI (Doenças do Sistema Nervoso) – código: G00-G99, especificamente na subcategoria mais aproximada ao termo, Paralisia Cerebral não especificada – código: G80.9. Após a avaliação médica, as crianças são encaminhadas para avaliação global, na qual a equipe multidisciplinar, composta por fisioterapeuta, fonoaudiólogo, psicólogo e terapeuta ocupacional, faz a observação clínica direta, descreve o seu desenvolvimento, discute o caso com o médico e define se a criança se beneficiará de intervenção e quais as terapias necessárias. Todas as crianças admitidas na AACD são submetidas a uma avaliação clínica inicial e outra final, quando atingem critério para alta. As avaliações são eminentemente clínicas, sem uso de testes padronizados. As crianças com ADNPM admitidas na AACD geralmente apresentam atraso motor, frequentam atendimentos semanais na fisioterapia, hidroterapia e terapia ocupacional até adquirirem marcha típica, quando recebem alta dos atendimentos. Apesar de a AACD/MG não oferecer programa de acompanhamento longitudinal para essas crianças, por não apresentarem deficiência física, elas podem retornar à Instituição para receber orientações ou ser submetidas às terapias com objetivos específicos, porém em curto prazo. Não existe programa específico de acompanhamento para essa população. Os retornos ficam a critério dos pais.

Nesse contexto, as crianças elegíveis para o estudo foram identificadas por meio de levantamento dos prontuários com o código G80.9, da CID-10, referente a agosto de 2001 a agosto de 2009, no setor de arquivo da AACD/MG. A listagem inicial continha 329 prontuários, que foram triados, segundo os critérios de inclusão e exclusão, até se chegar aos 45 participantes do estudo, como ilustrado na figura 1. Como critérios de inclusão, foram considerados: crianças de ambos os gêneros, nascidas entre janeiro de 2003 e abril de 2006, residentes em Uberlândia (MG), com diagnóstico de ADNPM, que apresentaram intercorrências pré/peri/pós-natal, com ausência de alterações neurológicas e/ou ortopédicas evidentes, má-formação e deficiências visuais ou auditivas. Só foram incluídas no estudo crianças que apresentaram, na última avaliação médica, marcha típica, as que tiveram alta dos atendimentos terapêuticos, com frequência em escola regular e cujos pais ou responsáveis assinaram termo de consentimento livre e esclarecido (TCLE), autorizando a participação no estudo. Foram excluídas as crianças que mudaram de diagnóstico para paralisia cerebral, distrofia muscular, autismo, retardo mental ou síndromes e também aquelas que permaneceram com o diagnóstico de ADNPM, mas apresentavam alterações neurológicas e/ou ortopédicas evidentes, má-formação, deficiências visuais ou auditivas.

**Figura 1:** Triagem dos prontuários para selecionar os participantes do grupo ADNPM.

As crianças com desenvolvimento típico foram recrutadas nas mesmas escolas dos casos provenientes da AACD/MG. O recrutamento de cada criança do grupo típico foi feito na mesma sala de aula de cada criança com diagnóstico de ADNPM. Os autores, após receber o consentimento dos pais da criança com ADNPM, foram à escola e fizeram o contato com o professor(a). Cada professor(a) foi solicitado(a) a identificar, pela lista de presença, alunos de mesmo sexo e idade daquele com diagnóstico de ADNPM. Localizadas as crianças, foi feito sorteio de uma delas e enviada carta convite (TCLE) aos pais/responsável. Junto com o TCLE, os pais/responsável responderam a um breve questionário sobre a história do desenvolvimento a fim de garantir que a criança apresentava perfil para o grupo. Só foram avaliadas as crianças cujo TCLE retornou assinado e com o questionário respondido pelos pais/responsável. Caso contrário, o(a) professor(a) sorteava outro aluno. Foram excluídas as crianças que apresentaram, no questionário, diagnóstico de transtornos neurológicos ou genéticos específicos e fatores de risco, tais como prematuridade e/ou baixo peso ao nascimento, deficiências auditivas e visuais, bem como problemas ortopédicos (fratura de membros inferiores e outros), uso contínuo de anticonvulsivantes, doença prolongada nos três meses anteriores ao teste, história de repetência e dificuldade escolar que necessitasse de suporte pedagógico ou algum tipo de terapia especializada (fisioterapia, fonoaudióloga, psicologia, terapia ocupacional).

Os seguintes instrumentos foram usados na pesquisa:

- **Roteiro semiestruturado** para extração de dados do prontuário das crianças com ADNPM provenientes da AACD/MG. Informações sobre a história da criança (idade gestacional, peso ao nascer e condições neonatais), diagnóstico médico ao entrar na instituição e aquele registrado na última consulta feita na unidade, dados dos pais/responsável (escolaridade materna e paterna, renda familiar), dados da escola (nome, endereço, telefone, professora e série), além de aspectos da reabilitação (avaliação inicial e final da criança feita pela equipe de reabilitação e as terapias feitas durante o período de intervenção). Como a avaliação inicial e final de cada criança é feita de forma descritiva, as informações foram categorizadas em três componentes: (a) motor, para a avaliação do setor de fisioterapia; (b) atividade, para o setor de terapia ocupacional; e (c) participação, para a avaliação do setor de psicologia. A informação de cada componente foi codificada de forma a indicar se o nível de desenvolvimento da criança era atrasado, suspeito ou adequado. A coleta dos dados dos prontuários foi feita por duas pesquisadoras que fizeram treinamento prévio com 20 prontuários e se obteve índice de concordância Kappa considerado de bom ajuste (0,63 a 0,79).
- **Classificação do desempenho acadêmico**: como na faixa de idade avaliada não há atribuição de notas, com base nas informações obtidas com a professora sobre o conceito que a criança recebeu nas capacidades trabalhadas ao longo do ano escolar, foi criada uma classificação. Conforme informação obtida nas escolas, as crianças são avaliadas ao longo do ano pela professora, que tanto atribui conceitos à aquisição de habilidades como identifica o nível em que elas se encontram na aprendizagem da leitura e escrita (alfabético, silábico-alfabético, silábico e pré-silábico). Essas informações foram combinadas e categorizadas da seguinte forma: (a) nível 1 – aquisição consolidada das habilidades esperadas para o ano escolar – a criança apresentava nível alfabético e/ou conceito A; (b) nível 2 – aquisição em desenvolvimento das habilidades esperadas para o ano escolar – nível silábico-alfabético ou silábico e/ou conceito B; e (c) nível 3 – sem domínio das habilidades esperadas para o ano escolar – apresentou nível pré-silábico e/ou conceito.
- **Movement Assessment Battery for Children – MABC-2**:^8^ teste padronizado para identificar problemas de coordenação motora em crianças e adolescentes de quatro a 16 anos, dividido em três áreas: destreza manual, agarrar e lançar e equilíbrio. A soma da pontuação de cada categoria fornece um escore padronizado e a soma das três categorias fornece o escore total, que é convertido em percentil. O ponto de corte ≤15% indica possível prejuízo motor e pontuação ≤5% indica déficit motor definitivo. Considerou-se que crianças com pontuação ≤5% apresentavam problemas de coordenação motora ou sinais de transtorno do desenvolvimento da coordenação (TDC), percentil de 6 a 15, casos suspeitos, e crianças com percentil acima de 15 foram consideradas como apresentando desempenho motor normal.
- **School Function Assessment – SF**A:^9^ questionário para avaliar o desempenho funcional e a participação de crianças de 5 a 12 anos no ambiente escolar, consiste de três partes: participação nos diferentes ambientes escolares, auxílio nas tarefas e desempenho de atividades. Os escores brutos da SFA são convertidos em escala de zero a 100, o último o ponto mais alto ou grau pleno de funcionamento na área avaliada. Os resultados da SFA podem ser interpretados de duas formas, nível básico e avançado. Para o presente estudo foi usado o nível básico, que informa se a função da criança em um ambiente da escola está dentro do esperado para crianças da mesma idade e ano escolar.
- **Inventário de Recursos do Ambiente Familiar - RAF**:^10^ questionário usado para avaliar recursos do ambiente familiar, dividido em três domínios: recursos materiais, atividades que sinalizam estabilidade na vida familiar e práticas parentais. Para obter pontuação relativa, em 10 pontos, foi usada a seguinte fórmula: *pontuação bruta/ pontuação máxima do tópico ×10*, em que pontuação bruta é o número de itens assinalados e pontuação máxima corresponde ao número total de itens, exceto nos tópicos 8-10, que têm pontuação específica. A pontuação relativa é útil para comparar a pontuação entre os itens do inventário.

Os dados foram coletados de janeiro de 2010 a janeiro de 2012. Todas as crianças que participaram deste estudo foram avaliadas pelo teste MABC-2, pelos questionários SFA e RAF e foi feita classificação do desempenho escolar com base nos relatórios das professoras. O MABC-2 é um dos testes mais usados em pesquisa para o diagnóstico de TDC e foi usado no presente estudo, para avaliar o desempenho motor das crianças. O MABC-2 tem bons índices de confiabilidade teste-reteste (0,75) e entre-examinadores (0,70),^11^^,^ 12 é um teste usado em diferentes países e há evidência da validade dos escores para crianças brasileiras.^13^ Pelo fato de o MABC-2 ser uma prova de desempenho, antes da coleta de dados foi verificada a confiabilidade entre examinadores e obteve-se o índice de 0,80 (Correlação Intraclasse).

A SFA vem sendo usada no Brasil e é um questionário de fácil aplicação, com conteúdo considerado adequado para documentar o perfil funcional de crianças na idade escolar. Apesar de não ser um questionário validado e normatizado para crianças brasileiras, estudos americanos dão suporte à validade e confiabilidade do instrumento.^14^ O RAF tem se mostrado útil para distinguir características do ambiente familiar de crianças com diferentes níveis de desempenho escolar e problemas de comportamento e apresenta parâmetros adequados de confiabilidade teste-reteste (0,92 a 1,00), além de boa consistência interna (0,84).^15^ Apesar de não ter ponto de corte, esse inventário vem sendo usado no Brasil na comparação entre grupos de crianças.

Todas as crianças residiam em Uberlândia (MG) e todas foram avaliadas pela primeira autora, previamente treinada para a aplicação dos testes e questionários. O questionário SFA e informações sobre o desempenho escolar (conceito) foram aplicados com o(a) professor(a) da criança na escola. Nessa visita, a primeira autora já explicava ao(à) professor(a) sobre o sorteio de uma criança da mesma sala de aula da criança sem ADNPM. Tais crianças foram avaliadas posteriormente, mediante o TCLE assinado.

A primeira autora visitou 35 escolas (20 da rede municipal, oito estaduais, seis particulares e uma federal), pois das 45 crianças do grupo ADNPM apenas dez estudavam na mesma escola. Para a entrada da pesquisadora nas escolas municipais, foi necessário obter consentimento da Prefeitura de Uberlândia (Secretaria de Educação). Nas demais (estadual, federal e particular), foi feito contato individual. Em todas as escolas foi apresentada a documentação que comprovava a permissão dos pais das crianças com ADNPM (TCLE) e a aprovação do estudo, pelo Comitê de Ética e Pesquisa da AACD (n° 09/2010) e Comitê de Ética em Pesquisa da Universidade Federal de Minas Gerais (COEP/UFMG n° ETIC 0482.0.203.000-10).

Para a análise de dados usou-se o Statistical Package for the Social Sciences (SPSS) para Windows, versão 17.0. A descrição dos grupos foi feita por meio de medidas de tendência central (média e desvio-padrão) ou frequência. Foi aplicado o teste de normalidade de Shapiro-Wilk, que detectou que a maioria das variáveis seguia distribuição normal, e optou-se pelo uso de testes paramétricos. A análise de variância (Anova) foi usada para a estatística inferencial com vistas a identificar possíveis diferenças entre os grupos ADNPM e típico nas variáveis quantitativas de desempenho motor, recurso ambiental e participação no ambiente escolar. Para a variável categórica desempenho no conteúdo acadêmico foi usado o teste binomial para duas proporções. Para todas as análises foi considerado nível de significância ≤0,05.

## Resultados

Das 65 crianças com diagnóstico de ADNPM provenientes da AACD/MG, 45 (69,3%) participaram do estudo e o restante (20; 30,7%) foram dados perdidos por desistência, mudança de cidade e crianças não localizadas. Assim sendo, o grupo ADNPM foi constituído por 45 crianças com diagnóstico de ADNPM e o grupo típico por 45 crianças com desenvolvimento típico, selecionadas por amostragem não aleatória, pareada por gênero, idade e renda familiar. A tabela 1 apresenta informações descritivas dos grupos.

**Tabela 1:** Características das crianças com atraso do desenvolvimento neuropsicomotor (ADNPM) e com desenvolvimento normal (DN), na idade escolar

Características dos participantes		Grupos	
		Com atraso	Típico
*Sexo* *			
	Masculino	26 (57,8)	26 (57,8)
	Idade (meses) **	95,8±7,7 a	95,4±7,6 a
*Renda familiar* *			
	Abaixo de 3 SM	27 (60%) b	26 (57,7%) b
	Entre 3 e 5 SM	7 (15,6%) c	12(26,7%) c
	Acima de 5 SM	11 (24,4%) d	7 (15,6%) d
	Idade gestacional	35,49 (4,88) **	>38 semanas
	Peso médio ao nascer **	2.289±1.116,17	3.086±500,37
*Escolaridade materna* *			
	Superior	11 (24,4)	13 (28,9)
	Médio	20 (44,4)	25 (55,6)
	Fundamental	13 (29)	6 (13,3)
	Analfabeto	1 (2,2)	1(2,2)

a *p* =0,794.

b *p* =0,830.

c *p* =0,197.

d *p* =0,292.

* Frequência (porcentagem) de crianças em cada categoria.

** Média±desvio‐padrão.

SM, Salário mínimo.n=45 em cada grupo.

No grupo ADNPM, 23 (51,1%) crianças nasceram prematuramente, com idades gestacionais de 24 a 36 semanas. No período neonatal, 17 (37,8%) apresentaram icterícia, 13 (28,9%) tiveram crises convulsivas, 14 (31,1%) relataram necessidade de suplementação de oxigênio e 12 (26,7%) apresentaram sinais de hipóxia perinatal. O grupo típico foi constituído por crianças nascidas a termo, sem registro de intercorrências neonatais relevantes.

As crianças do grupo ADNPM tinham comumente como características iniciais do desenvolvimento atraso motor (24; 55,6%) e nível de desenvolvimento suspeito nas áreas de atividade (27; 60%) e participação (27; 60%). O término da intervenção multidisciplinar, que durou em média 2,61±1,96 anos, se deveu, principalmente, ao fato de o desenvolvimento motor ter sido considerado adequado para a idade (41; 91,1%).

Houve diferença média entre os grupos com significância estatística em todas as áreas do MABC-2 (tabela 2). O grupo ADNPM obteve desempenho inferior em todos os domínios do teste, com a maioria das crianças desse grupo (28; 62,2%) apresentando dificuldade motora e quatro (8,9%) escolares com risco para dificuldade motora. No grupo típico, cinco (11,1%) crianças tiveram dificuldade motora e seis (13,3%) apresentaram risco para dificuldade motora.

**Tabela 2:** Dados comparativos dos percentis de desempenho motor para os grupos com atraso e típico

MABC‐2	Média±DP			Mínimo‐Máximo		
	Com atraso	Típico		Com atraso	Típico	*p* ‐valor a
Destreza manual	20,1±26,2	48,7±31,2		0,5–98	2–99,9	<0,001
Lançar e agarrar	19,8±21,2	30±23,5		0,5– 91	1– 91	0,034
Equilíbrio	14±21,8	33,2±26		0,1– 95	2– 99	<0,001
Motor total	13,2±21,6	34,3±27		0,1– 91	2– 98	<0,001

a Anova.

MABC‐2, Movement Assessment Battery for Children; DP, desvio‐padrão; n, 45 em cada grupo.

Na SFA, as crianças do grupo ADNPM tiveram escores médios mais baixos em todos os itens avaliados do questionário em relação ao grupo típico. Apenas no quesito *autocuidado* (*p*=0,183) não houve diferença entre os grupos. Enquanto 30 crianças do grupo típico (66,7%) mostraram participação efetiva no ambiente escolar, com ajuda nas tarefas cognitivas e comportamentais similar à oferecida aos colegas do mesmo ano, apenas 10 (22,2%) crianças com atraso apresentaram o mesmo desempenho. Em relação às atividades acadêmicas, o grupo típico apresentou desempenho consistente e superior em todas as tarefas, quando comparado com grupo ADNPM. As crianças do grupo ADNPM apresentaram desempenho limitado principalmente nas atividades que exigiam interação positiva (26; 57,8%), controle do comportamento (26; 57,8%) e conclusão de tarefas (28; 62,2%) (tabela 3).

**Tabela 3:** Dados comparativos da pontuação no questionário de participação escolar para os grupos com atraso e típico

SFA		Média±DP			Mínimo‐Máximo		
		Com atraso a	Típico a		Com atraso	Típico	*p* ‐valor b
*Parte I Participação*							
	Participação no ambiente escolar	80±15,8	94,7± 0,6		54‐100	70‐100	<0,001
*Parte II Auxílio nas tarefas*							
	Cognitivas e comportamentais	75,5±23,3	90,5±12,8		0‐100	53‐100	<0,001
*Parte III Desempenho de atividades*							
	Uso de materiais	84,7±17,4	95,3±8,9		54‐100	68‐100	<0,001
	Trabalho escrito	77,5±23,5	91,8±11,2		15‐100	64‐100	<0,001
	Comunicação funcional	82,0±20,3	93,2±12,7		30‐100	49‐100	0,002
	Memória e compreensão	80,7±19,7	93,3±15,0		39‐100	27‐100	0,001
	Segurança	87,0±20,4	98,2±7,6		40‐100	53‐100	0,001
	Autocuidado	92,9±16,9	96,9±10,1		28‐100	55‐100	0,183
	Interação positiva	73,3±22,0	90,7±11,7		15‐100	54‐100	<0,001
	Controle do comportamento	65,8±29	85,9±15,9		0‐100	48‐100	<0,001
	Seguimento de regras	76,4±24,0	91,8±12,4		0‐100	55‐100	<0,001
	Obediência às ordens de adultos	78,0±22,0	87,7±17,8		29‐100	10‐100	0,024
	Comportamento e conclusão da tarefa	71,4±21,9	89,7±11,8		28‐100	59‐100	<0,001

a Dado médio bruto transformado para uma escala de zero a 100.

b Anova.

SFA, School Function Assessment; DP, desvio‐padrão; n, 45 em cada grupo.

Com relação ao desempenho acadêmico, houve diferença entre os grupos, com significância estatística nos níveis I (*p*=0,001) e II (*p*=0,008). A maior parte (38; 84,5%) das crianças do grupo típico apresentou domínio no conteúdo acadêmico (nível I – alfabético e/ou conceito ótimo) e o restante (6; 13,3%, nível II – silábico-alfabético ou silábico, conceito bom; 1; 2,2%, nível III – pré-silábico e/ou conceito regular) em fase de desenvolvimento. Já no grupo ADNPM, grande parte (24; 53,3%) alcançou nível I e nível II (17; 37,8%), com apenas quatro (8,9%) crianças ainda com conceito regular.

Como indicado na tabela 4, apesar de existir diferença nas médias entre os grupos nos itens do RAF, apenas dois indicadores atingiram significância estatística (*p*≤0,05). No grupo ADNPM, as crianças faziam menos atividades no tempo livre e compartilharam menos atividades com os pais em casa.

**Tabela 4:** Dados comparativos do inventário Recursos do Ambiente Familiar (RAF) para os grupos com atraso e típico

RAF	Pontuação bruta Média±DP			Pontuação relativa a Média±DP		
	Com atraso	Típico		Com atraso	Típico	*p* ‐valor b
Diversidade de atividades no tempo livre	3,8±1,2	4,4±0,9		6,2±1,9	7,4±1,6	0,003
Passeios feitos nos últimos 12 meses	7,4±2,3	8,2±2,9		4,1±1,3	4,9±1,6	0,121
Atividades programadas regulares	1,3±1,4	1,8±1,9		1,6±1,7	2,2±2,4	0,186
Atividades com os pais em casa	7,4±2,2	8,8±,6		7,4±2,2	8,8±1,6	0,001
Brinquedos e outros materiais	13,2±3,2	13,4±3,4		7,3±1,8	7,4±1,9	0,775
Diversidade de revistas e jornais	4,9±2,8	5,3±2,3		5,4±3,1	5,9±2,5	0,430
Diversidade de livros	5,5±1,6	5,4±1,6		6,8±2,0	6,7±2,0	0,809
Supervisão do trabalho escolar	11,6±2,4	12,6±2,2		6,4±1,3	7,0±1,2	0,058
Rotina diária com horários definidos	10,9±3,9	10,6±3,5		6,8±2,5	6,6±2,2	0,720
Momentos em que a família se reúne	8,3±2,4	8,7±2,2		6,9±2,0	7,3±1,8	0,368
RAF total	74,6±10,6	79,2±13,6		6,0±0,9	6,4±1,1	0,081

a Pontuação relativa: dado médio bruto transformado para uma escala de zero a 10

b Anova, pontuação relativa.

DP, desvio‐padrão; n, 45 em cada grupo.

## Discussão

Apesar de o termo ADNPM ser muito usado na literatura brasileira, pouco se sabe sobre o desfecho dessas crianças. O presente estudo demonstra que crianças com diagnóstico de ADNPM recebido até os dois anos apresentam, na idade escolar, limitações motoras, restrições no desempenho de atividades escolares, baixa participação no contexto escolar e desempenho funcional significativamente inferior ao de crianças sem história de atraso. Embora as crianças persistam com atrasos, o desfecho funcional foi melhor do que o motor, o que sugere a possibilidade de adaptação, o que é consistente com a perspectiva da CIF^7^ de que a relação entre a função motora e a participação não é linear. Estímulos ambientais, que podem atuar como fator de proteção, não parecem ter influenciado esses resultados.

Sabe-se que os fatores de risco para o atraso são múltiplos e o acúmulo de condições pode determinar maior impacto no desenvolvimento da criança.^16^^,^ 17 As crianças do grupo ADNPM incluídas neste estudo eram provenientes de famílias de baixa renda, que recebiam, em sua maioria, menos de três salários mínimos e com boa parte das mães com escolaridade no nível médio. Além disso, 51,1% das crianças do grupo ADNPM tinham histórico de prematuridade e baixo peso ao nascimento, além de intercorrências neurológicas neonatais. Embora não se possa excluir a influência de outros fatores não investigados no desfecho do grupo ADNPM, o risco biológico, representado especialmente pela prematuridade, foi decisivo sobre os demais fatores. Como discutido por alguns autores,^18^^,^ 19 apesar do baixo investimento na determinação etiológica, muitos estudos indicam os fatores biológicos como determinantes da maioria dos casos de atraso. No estudo de Srour et al.,^20^ por exemplo, no qual foi investigada a causa do atraso por meio de exames clínicos e laboratoriais, identificou-se a etiologia de 77% dos casos. A malformação cerebral, a encefalopatia hipóxico-isquêmica e as anormalidades cromossômicas foram as causas mais frequentes.

A alta frequência (62,2%) de alterações motoras na idade escolar encontrada no grupo ADNPM, corrobora a literatura. Metanálise de Willians et al.,^21^ com estudos de escolares nascidos prematuramente, indica prevalência de até 40,5% de alterações motoras *vs*. 6% na população geral. Embora o grupo típico também inclua crianças com dificuldades motoras (11,1%), a frequência foi próxima à esperada para a população geral, assim como observado no estudo de Goyen e Lui,^22^ que, ao avaliar crianças pré-termo e a termo com o teste MABC-2, encontraram prevalência de déficit motor de 42% nos pré-termo e 8% nos nascidos a termo.

Quanto ao desempenho escolar, medido pelo domínio dos conteúdos acadêmicos conforme avaliação do(a) professor(a), observou-se que pouco mais da metade (53,3%) das crianças do grupo ADNPM obteve conceito ótimo, o que indica avanço no processo de alfabetização. É interessante notar que mesmo na presença de alterações motoras, conforme comentado anteriormente, essas crianças conseguiram atingir domínio no conteúdo acadêmico. Apesar de as crianças persistirem com atraso, o desfecho funcional foi melhor do que o motor. Isso sugere que as crianças são capazes de se adaptar ou que é possível modificar o ambiente para facilitar a participação e a aprendizagem.^5^

As crianças do grupo ADNPM, no entanto, tiveram pior pontuação em todas as áreas de participação escolar da SFA. Apenas 22,2% delas *vs*. 66,7% no grupo típico. Participaram de maneira efetiva do ambiente escolar, sem necessitar de ajuda extra nas tarefas cognitivas e comportamentais. Shevell et al.^5^ também encontraram que apenas 17% das crianças com atraso participavam na sala de aula sem ajuda. Possivelmente, o atraso motor, como registrado no presente estudo, teve mais impacto no desempenho das atividades necessárias para participação na sala de aula (ex.: manejo de materiais, trabalho escrito) do que no desempenho acadêmico medido pela alfabetização, que não requer necessariamente o componente motor.

Dentre as limitações do estudo estão o uso de testes importados, sem pontos de corte completamente validados para a população brasileira; o uso do relatório do(a) professor(a) para classificar o desempenho acadêmico; e por se tratar de uma amostra de conveniência. Testes estrangeiros são usados rotineiramente em estudos brasileiros. O MBC-2 é de validação recente.^13^ Além disso, houve o cuidado de coletar dados comparativos. Como muitas crianças no grupo ADNPM apresentaram problemas na alfabetização, seria importante a avaliação de linguagem, o que deve ser incluído em estudos futuros. O relato do(a) professor (a) foi usado em outros estudos, como o de Pritchard et al.,^23^ que, ao comparar a avaliação qualitativa do professor com medidas padronizadas, encontraram que o relato detectou duas a três vezes mais crianças propensas a apresentar dificuldades escolares. Recomenda-se que estudos futuros saiam do contexto do centro de reabilitação para incluir população mais ampla, de postos de saúde, para se investigar o desfecho de casos mais leves e variados de ADNPM, pois pode proporcionar poder estatístico necessário na detecção de diferenças nos fatores ambientais.

Este estudo, desenvolvido dentro de um cenário clínico, ressalta a importância de reconhecer a necessidade de acompanhamento longitudinal do desenvolvimento das crianças que recebem diagnóstico precoce de ADNPM, pois, para muitas delas, o atraso é um sinalizador de condições futuras, que devem ser mais bem monitoradas e diagnosticadas, assim que a idade permitir. O acompanhamento dever ser feito pelo menos até a idade escolar para identificar as necessidades à medida que surgem e minimizar problemas na idade escolar e vida adulta. Avaliações periódicas podem auxiliar os profissionais e pais a entenderem o que está ocorrendo, de forma a dar suporte adequado à criança até a definição do “diagnóstico final”, uma vez que o termo ADNPM deve funcionar apenas como diagnóstico temporário.
